# Anticonvulsant and Toxicological Evaluation of Parafluorinated/Chlorinated Derivatives of 3-Hydroxy-3-ethyl-3-phenylpropionamide

**DOI:** 10.1155/2016/3978010

**Published:** 2016-02-24

**Authors:** Osvaldo Garrido-Acosta, Sergio E. Meza-Toledo, Liliana Anguiano-Robledo, Marvin A. Soriano-Ursúa, José Correa-Basurto, Asghar Davood, Germán Chamorro-Cevallos

**Affiliations:** ^1^Facultad de Estudios Superiores Zaragoza, Universidad Nacional Autónoma de México, 15500 México City, DF, Mexico; ^2^Laboratorio de Quimioterapia Experimental, Escuela Nacional de Ciencias Biológicas, Instituto Politécnico Nacional, 11350 México City, DF, Mexico; ^3^Laboratorio de Farmacología Molecular, Sección de Estudios de Posgrado e Investigación, Escuela Superior de Medicina, Instituto Politécnico Nacional, 11340 México City, DF, Mexico; ^4^Departamento de Fisiología, Escuela Superior de Medicina, Instituto Politécnico Nacional, 11340 México City, DF, Mexico; ^5^Laboratorio de Modelado Molecular y Diseño de Fármacos, Escuela Superior de Medicina, Instituto Politécnico Nacional, 11340 México City, DF, Mexico; ^6^Department of Medicinal Chemistry, Pharmaceutical Sciences Branch, Islamic Azad University, Tehran 19419, Iran; ^7^Laboratorio de Toxicología Preclínica, Escuela Nacional de Ciencias Biológicas, Instituto Politécnico Nacional, 11350 México City, DF, Mexico

## Abstract

Although the anticonvulsant activity of 3-hydroxy-3-ethyl-3-phenylproionamide (HEPP) is well-known, its use is limited by the pharmacotoxicological profile. We herein tested its fluorinated and chlorinated derivatives (F-HEPP and Cl-HEPP) with two seizure models, maximal electroshock seizures (MES), and intraperitoneal pentylenetetrazole (PTZ) administration. Neurotoxicity was examined via the rotarod test. With in silico methods, binding was probed on possible protein targets—GABA_A_ receptors and the sodium channel Nav1.2. The median effective doses (ED_50_) of HEPP, F-HEPP, and Cl-HEPP in the MES seizure model were 129.6, 87.1, and 62.0 mg/kg, respectively, and 66.4, 43.5, and in the PTZ seizure model 43.5 mg/kg. The HEPP-induced neurotoxic effect, which occurred at twice the ED_50_ against MES (*p* < 0.05), did not occur with F-HEPP or Cl-HEPP. Docking studies revealed that all tested ligands bound to GABA_A_ receptors on a site near to the benzodiazepine binding site. However, on the sodium channel open pore Nav1.2, R-HEPP had interactions similar to those reported for phenytoin, while its enantiomer and the ligands F-HEPP and Cl-HEPP reached a site that could disrupt the passage of sodium. Our results show that, as anticonvulsant agents, parahalogen substituted compounds have an advantageous pharmacotoxicological profile compared to their precursor.

## 1. Introduction

Epilepsy refers to brain function disorders characterized by periodic and unpredictable occurrences of seizures [1]. A seizure is a transient change in the neuronal populations of the central nervous system that triggers synchronous disorders and paroxysmal discharges [2]. Epidemiological studies have indicated that the incidence of this disease has increased in the last 40 years [3]. Currently, over 50 million people worldwide have epilepsy [4].

No single antiepileptic drug (AED) has been shown to be the most effective for the treatment of epilepsy and all have side effects [5]. Between 70% and 80% individuals are successfully treated with one of the AEDs now available. However, 20–30% of patients have either intractable or uncontrolled seizures or suffer significant adverse side effects after taking medication [6]. Success or failure of treatment primarily depends on the etiology of the seizure disorder.

Due to the large number of patients without effective treatment, there is an ongoing search for new anticonvulsant drugs. Some recent studies suggest advances in this effort, using techniques of medicinal chemistry and pharmacology to design new compounds or modify those already in use. Additionally, light has been shed on innovative mechanisms of action [7–11].

**Figure 1:**
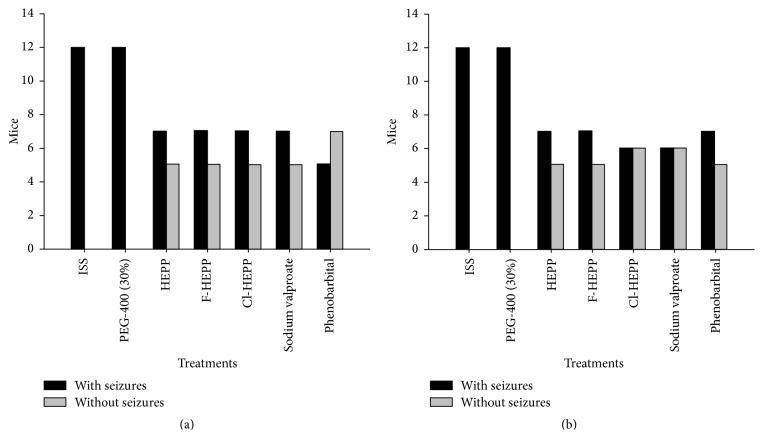
The effect of the tested compounds on two seizure models. (a) The effect of administering the ED_50_ against maximal electroshock seizures: HEPP = 129.6 mg/kg, F-HEPP = 87.1 mg/kg, Cl-HEPP = 62.0 mg/kg, sodium valproate = 261.2 mg/kg, and phenobarbital = 16.9 mg/kg; (b) the effect of administering the ED_50_ against pentylenetetrazole seizures: HEPP = 66.4 mg/kg, F-HEPP = 43.5 mg/kg, Cl-HEPP = 43.5 mg/kg, sodium valproate = 159.7 mg/kg, and phenobarbital = 12.9 mg/kg. *n* = 12. Black bar, mice with seizures; grey bar, mice without seizures; ISS, isotonic saline solution (NaCl 0.09%); PEG-400 (30%), polyethyilenglicol-400 at 30% with ISS. For the hypothesis test, the percentage of protection was 50% in order to determine the proportion response of the antiepileptic drugs. According to the *Z*-test, there was no statistically significant difference between mice protected against seizures with antiepileptic drugs and unprotected mice.

In 1990, 3-hydroxy-3-ethyl-3-phenylpropionamide (HEPP, Figure 1(a)) was synthesized and tested as an anticonvulsant agent, showing promising effects [12]. In tests of anticonvulsant activity against maximal electroshock seizures (MES) and pentylenetetrazole (PTZ), HEPP had a median effective dose (ED_50_) of 144 and 63 mg/kg, respectively. The median toxic dose (TD_50_), evaluated by the rotarod test, was 214 mg/kg [12, 13].

Because HEPP is effective against PTZ and MES seizures and based on data from preliminary structural analysis, it has been suggested that its biological activity may be due to the modulation of GABA receptors as well as the blockade of the sodium channel Nav1.2 [14]. What is actually known about the mechanism of action is that HEPP moves flunitrazepam of the benzodiazepine binding site and t-butyl-bicyclophosphorothioate of the picrotoxin binding site in GABA_A_ receptors [15]. Also, results from nigra slices of rat brains have revealed that HEPP does not inhibit the release of GABA produced by electrical stimulation [14]. Rather, it significantly reduces the inhibitory effect of GABA on the release of [^3^H]-GABA via electrical stimulation or in the presence of potassium chloride and tiagabine, the latter being an inhibitor of GABA recapture. Moreover, when [^3^H]-GABA is stimulated electrically, HEPP reverses the inhibitory effect caused by the release of bicuculline and picrotoxin (GABA_A_ receptor antagonists) in substantia nigra slices from rat brains [14].

**Scheme 1:**
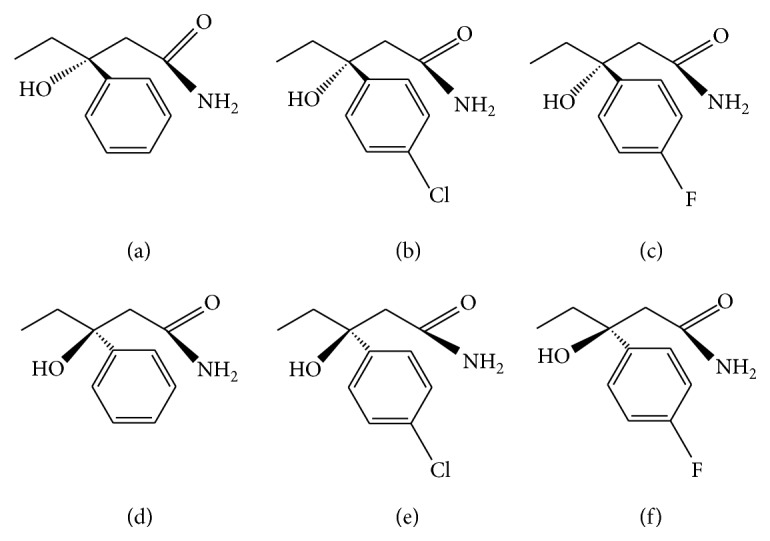
Chemical structure of compounds tested in this study. (a) 3(R)-3-Hydroxy-3-ethyl-3-phenylpropionamide, (R)-HEPP; (b) 3(R)-3-hydroxy, 3-ethyl, 3-(4′-chlorophenyl)propionamide, (R)-Cl-HEPP; (c) 3(R)-3-hydroxy, 3-ethyl, 3-(4′-fluorophenyl)propionamide, (R)-F-HEPP; (d) 3(S)-3-hydroxy-3-ethyl-3-phenylpropionamide, (S)-HEPP; (e) 3(S)-3-hydroxy, 3-ethyl, 3-(4′-chlorophenyl)propionamide, (S)-Cl-HEPP; (f) 3(S)-3-hydroxy, 3-ethyl, 3-(4′-fluorophenyl)propionamide, (S)-F-HEPP.

Some derivatives of HEPP have been synthesized and studied in order to obtain compounds with better anticonvulsant properties. Accordingly, 3-hydroxy, 3-ethyl, 3-(4′-fluorophenyl)propionamide (F-HEPP; Scheme 1(c)) and 3-hydroxy, 3-ethyl, 3-(4′-chlorophenyl)propionamide (Cl-HEPP; Scheme 1(b)) were synthesized by starting from HEPP and adding a fluorine or chlorine atom to the benzene ring in paraposition. These compounds were then tested for their ability to increase availability and potency in the brain when compared with HEPP [16, 17].

Regarding the pharmacological activity of these derivatives, scant data has been reported using a seizure model. The available results showed that compared to HEPP, both F-HEPP and Cl-HEPP have greater potency as an anticonvulsant agent against seizures induced by PTZ (100 mg/kg), calculating an ED_50_ of 43 and 20 mg/kg, respectively [16, 17]. Regarding the mechanism of action, even less is known about these derivatives than HEPP. It has been inferred that they have some mechanisms in common with the lead compound [17].

In order to compare HEPP and its fluorinated and chlorinated derivatives, we tested the anticonvulsant effects with two seizure methods and evaluated the neurotoxic effects with the rotarod test. In addition, docking studies of the target compounds were performed on GABA_A_ receptors as well as on the sodium channel Nav1.2 to determine the possible pharmacological mechanism.

## 2. Material and Methods

### 2.1. Animal Care and Use

We used male CD1 mice weighing 30 ± 3 g, acquired from the Vivarium of Hidalgo State University (in the city of Pachuca, Mexico). All of the mice were kept in cages with saw dust bedding in a room at 22–24°C, 40–50% relative humidity, light and dark cycles of 12:12 h, and food and water provided ad libitum. All of the experiments complied with the requirements and guidelines established by the Secretaría de Agrícultura, Ganadería, Desarrollo Rural y Pesca (SAGARPA), as established by the Mexican Official Standard (NOM-062-ZOO-1999) [18], which specifies the proper use, care, and management of laboratory animals. Furthermore, all of the experiments were conducted under the authorization and regulations of the Institutional Bioethics Committee at the Escuela Nacional de Ciencias Biológicas, Instituto Politécnico Nacional (Mexico).

### 2.2. Chemicals

Phenobarbital (PubChem CAS 50-06-6), sodium valproate (CAS 1069-66-5), hydrochloric acid (HCl), pentylenetetrazole (PTZ; CAS 54-95-5), and polyethylenglycol-400 were purchased from Sigma-Aldrich (St. Louis, MO, USA). A racemic mix of 3-hydroxy-3-ethyl-3-phenylpropionamide (HEPP), as well as its fluorinated and chlorinated derivatives (F-HEPP and Cl-HEPP), was synthesized and submitted to chemical characterization using previously described methods [7, 11, 12]. The compounds yielded had a purity of ≥99.5%. Phenobarbital was dissolved in a solution of 0.01 N HCl and sodium valproate and PTZ were dissolved in an isotonic saline solution (ISS) of NaCl. HEPP, F-HEPP, and Cl-HEPP were dissolved in polyethylenglycol-400 at 30% with ISS. The solutions were prepared and used the same day and the compounds were administered intraperitoneally (i.p.).

### 2.3. MES and PTZ Seizure Induction

Seizures were induced using MES with a rectangular pulse generator (Ugo Basile ECT Unit 7801). The pulse was applied with headphone electrodes covered with conductive gel placed on the ears of the mice. A pulse width of 0.5 ms was generated at a current of 20 mA and a frequency of 100 Hz and was applied for 200 ms. Abolition of the hind limb tonic extensor component was used as the end point in this test. Seizures were induced using PTZ by administering 126 mg/kg (i.p.). Suppression of clonic seizures and death was considered the end point.

### 2.4. Anticonvulsant Treatments

Sodium valproate and phenobarbital were administered i.p. 15 and 45 min before the induction of seizures, respectively. HEPP, F-HEPP, and Cl-HEPP were administered once to each animal (at 0.01 mL/g) 30 min before the induction of seizures.

### 2.5. ED_50_ Determination

Lorke's method modified by Garrido-Acosta et al. [19] was used to obtain the ED_50_ in the MES and PTZ seizure models. These values were validated using statistical studies.

### 2.6. Rotarod Test

To determine the neurotoxic effect (motor disparity) of anticonvulsant compounds, we used a rotarod (rotamex) with a 29.3 mm diameter roller [20–22]. The test was conducted with the rotating roller accelerating from 0 to 21 rpm in 70 s, followed by a steady rate for an additional 110 s (total time = 180 s).

The mice that remained on the rotating roller for 180 s were selected for further testing, forming seventeen groups of 8 mice each. Each anticonvulsant compound was administered to 3 groups in the following doses: one group with the ED_50_ against PTZ seizures, one with the ED_50_ against seizures by MES, and one with twice the ED_50_ against seizures by MES. ISS was administered to one group and PEG-400 at 30% to another. The data are plotted as a percentage of time spent on the rotarod, considering 180 s as 100%.

### 2.7. Statistical Analysis

A hypothesis test for rations was applied to validate that the fraction of mice protected was 50% in each anticonvulsant treatment. Furthermore, a* Z*-test for rations was applied to validate equality between the fraction of mice protected and not protected in the test, based on the results of the modified Lorke's method. The Wilcoxon test was used to analyze the results of the neurotoxic effects of the compounds in the rotarod test. For all statistical analysis, we used SigmaStat version 3.5 software. For graphs, we used SigmaPlot version 10.0. Statistical significance was considered at ^*∗*^ *p* < 0.05.

### 2.8. Computational Methods

#### 2.8.1. Molecular Structure and Structural Optimization of Ligands

All molecular structures used in this work (Scheme 1) were drawn with the Gaussian view 03 program and their chemical structures were optimized using Gaussian 03 via the AM1 [23–25] semiempirical methodology level of calculation to reduce the energy levels of the system to the global minimum as well as to ensure structural stability and an absence of steric clashes.

### 2.9. Receptor Selection

Since it is well-known that GABA_A_ receptors have a binding site at the benzazepine pharmacophore, we focused on this core scaffold and studied a benzodiazepine-like flunitrazepam, as reported elsewhere [26], with the aim of identifying a better binding site. For this initial validation, we loaded the optimized structure with AutoDockTools 1.5.2 [27] in order to prepare the input files for the docking studies. Hence, the target ligands were docked into the GABA_A_ receptor structure, as reported by Muroi et al. [28]. For the sodium channel open pore Nav1.2 study, we selected an open pore model that was developed based on the homology model of the crystal structures of potassium channels [29], prepared and validated as described elsewhere [30].

### 2.10. Docking Simulations

To prepare the receptors, hydrogen atoms were added using the PSFGEN program included in Visual Molecular Dynamics 1.8 [31], and then the entire receptor was minimized with 2000 steps using the CHARMM27 force field [32] implemented in Nanoscale Molecular Dynamics 2.6 [33], followed by geometrically optimizing the ligands with Gaussian 03, as described above. Afterwards, all possible flexible bonds were identified and the partial atomic charges of the ligands (Gasteiger-Marsili formalism) were calculated using AutoDockTools 1.5.6. For the receptors, the Kollman charges for all of the atoms were computed after using the polar hydrogen to evaluate the hydrogen bonding interactions. All of the other parameters were maintained at their default settings. The receptor exploration and binding site definitions were prepared employing a GRID-based procedure [34], using a 60 × 60 × 60 Å point grid with 0.375 Å spacing. The center was set at *x* = 14.210, *y* = 43.476, and *z* = 27.894 on GABA_A_ and at *x* = 8.066, *y* = 1.086, and *z* = −7.603 on the sodium channel. All of the docking simulations used the hybrid Lamarckian Genetic Algorithm [27], with an initial population of 100 and 1 × 107 evaluations. Docked orientations within a root mean square deviation (RMSD) of 0.5 Å were clustered together.

The lowest Gibbs free energy cluster returned for each compound docked in the receptor structure was used for further analysis. Interactions of the ligands with GABA_A_ and the sodium channel Nav1.2 structure were visualized using AutoDockTools 1.5.6. For validating purposes, the reference compound for these docking assays was flunitrazepam for the GABA_A_ receptor and the diphenylhydantoin (phenytoin) molecule for the sodium channel model.

## 3. Results

### 3.1. ED_50_ of HEPP, F-HEPP, and Cl-HEPP

The ED_50_ of anticonvulsants are shown for both seizure models, MES and PTZ, as is the 95% confidence limit of proportions expected with the administration of these values (Table 1).

### 3.2. Statistical Validation of the ED_50_ against MES and PTZ Seizures

The ED_50_ values of HEPP, F-HEPP, Cl-HEPP, sodium valproate, and phenobarbital protected 50% of the mice against MES seizures (Figure 1(a)) as well as 50% against PTZ seizures (Figure 1(b)).

### 3.3. Rotarod Test

**Figure 2:**
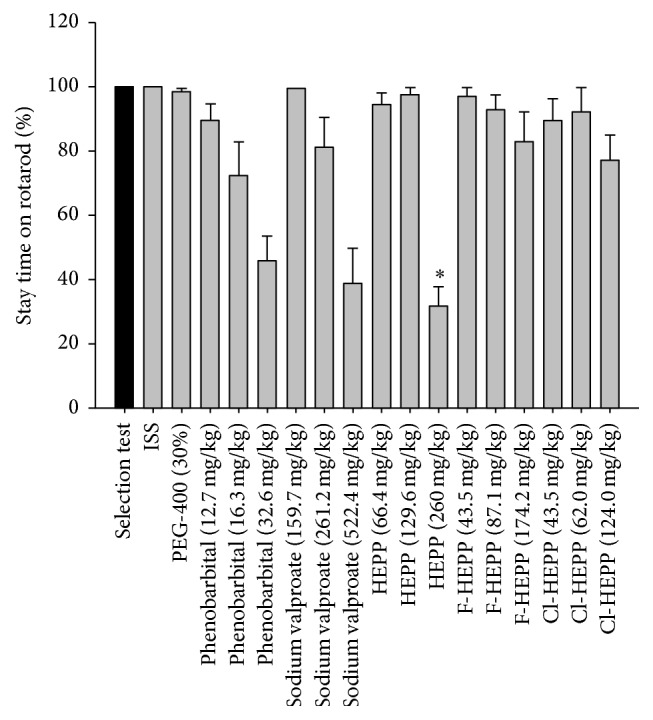
Effect on the rotarod test produced by the administration of different doses of HEPP, F-HEPP, Cl-HEPP, sodium valproate, and phenobarbital. *n* = 8. Black bar, selection test (180 s = 100%); grey bar, evaluation test (expressed as the median + standard error of the mean). The doses administered for each treatment were ED_50_ against PTZ < ED_50_ against MES < twice ED_50_ against MES. ISS, isotonic saline solution (NaCl 0.09%); PEG-400 (30%), polyethyilenglicol-400 at 30% with ISS. Kruskal-Wallis post hoc Dunn's, ^*∗*^statistically significant difference with respect to the selection test (*p* < 0.05).

The effect of the administration of HEPP, F-HEPP, Cl-HEPP, sodium valproate, and phenobarbital is shown as the duration of the rotarod test (Figure 2). Only at twice the ED_50_ of HEPP against MES seizures was there a statistical difference (*p* < 0.05) with respect to the selection test.

### 3.4. Docking Studies on GABA_A_

**Figure 3:**
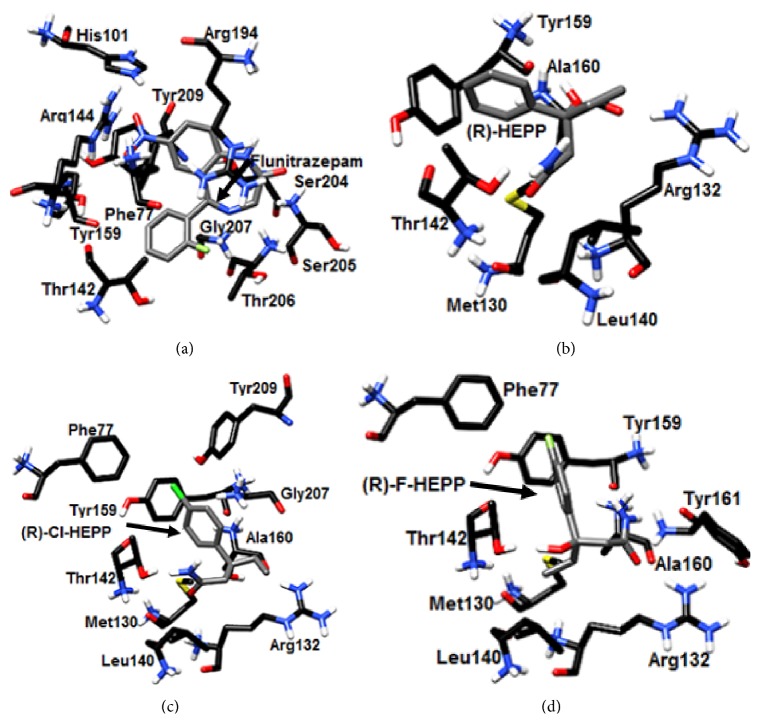
Mode of binding of (R)-HEPP, (R)-Cl-HEPP, and (R)-F-HEPP at the benzodiazepine binding site of the GABA_A_ receptor.

**Figure 4:**
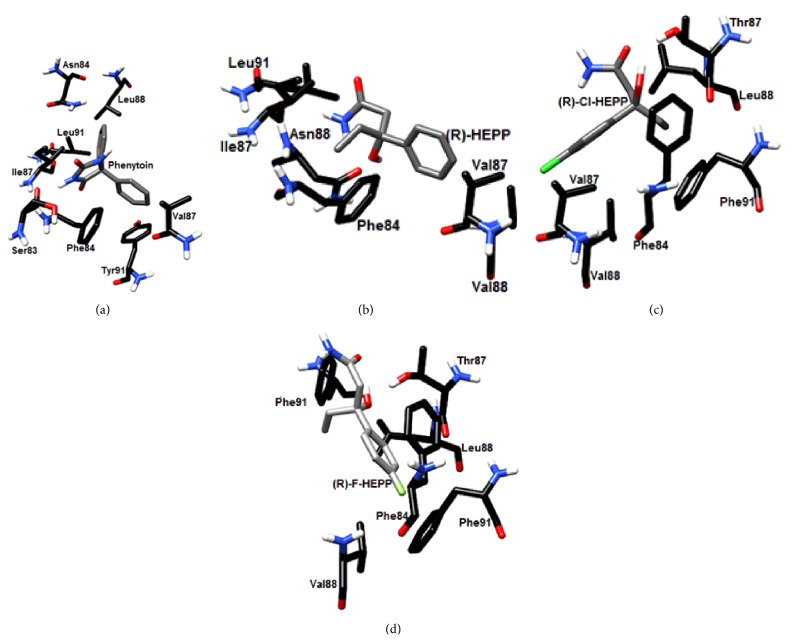
Mode of binding of phenytoin, (R)-HEPP, (R)-Cl-HEPP, and (R)-F-HEPP on the sodium channel Nav1.2.

The docking test yielded binding poses for the R and S absolute configurations, of HEPP, F-HEPP, and Cl-HEPP in the benzodiazepine binding site on GABA_A_. (Figure 3 and Supplementary Figure  1; see Supplementary Material available online at http://dx.doi.org/10.1155/2016/3978010). Each in the conformation of the highest score has two to four amino acids that interact the same as flunitrazepam, the ligand taken as reference compound. As putative key interactions, flunitrazepam produces *π*-cation interactions with the sidechain of Arg104 as well as aromatic interactions with Phe99, by means of hydrogen bonds with Asn110 and Van Der Waals interactions with the backbones of Arg135 and Val134 (Figure 4(a)). R-HEPP produces aromatic interactions with Phe169 and Trp123, hydrophobic interactions with Ala121 and Ile147, and hydrogen bonds with Thr146 (Figure 3(b)). R-Cl-HEPP produces aromatic interactions with Phe121, hydrophobic interactions with Ala121, and hydrogen bonds with the backbones of Ile147, Thr146, and Leu145 (Figure 3(c)). R-F-HEPP makes hydrogen bond interactions with the backbone of Asn128 and Thr95, and the OH of the compounds produces hydrogen bond interactions with sidechains of Asp97, Met130, Tyr161, and Met102 (Figure 3(d)).

S-HEPP produces aromatic interactions with Trp123, hydrophobic interactions with Leu145 and Ile147, and hydrogen bonds with Asp120 and Ser116 (Figure S1B). S-Cl-HEPP produces halogen hydrogen bonds with the backbone of Asn101 and Ser100, aromatic interactions with Tyr161, and hydrogen bonds with the backbone of Asn27 and Leu29 (Figure S1C). S-F-HEPP produces aromatic interactions with Phe169 and Trp123, hydrophobic interactions with Leu145, and hydrogen bonds with Asp120 (Figure S1D).

Supplementary Table  1 lists the amino acids involved in the interaction of HEPP, F-HEPP, and Cl-HEPP in its absolute R and S configurations in the benzodiazepine binding site, as well as those involved in the binding of flunitrazepam.

### 3.5. Docking Studies on the Sodium Channel Nav1.2

The docking test of the R and S absolute configurations is shown for HEPP, F-HEPP, and Cl-HEPP on sodium channel Nav1.2 (Figure 4 and Supplementary Figure  2). HEPP, F-HEPP, and Cl-HEPP (each in the conformation of highest docking score) have two or three amino acids that interact the same as phenytoin, the sodium channel blocker used as the reference.

On the 3D model of this sodium channel, phenytoin docks by means of hydrophobic interactions with Leu88, Leu99, and Ile87, aromatic interactions with Tyr91 and Phe84, and hydrogen bonds with Ser83 (Figure 4(a)). R-HEPP binds by means of aromatic interactions with Phe84, hydrophobic interactions with Val188, and hydrogen bonds with the backbones of Ile87 and Asn88 (Figure 4(b)). R-Cl-HEPP makes contact by means of aromatic interactions with Phe91 and Phe91 and hydrophobic interactions with Leu88 (Figure 4(c)). R-F-HEEP produces aromatic interactions with Phe84 and Phe91 and hydrophobic interactions with Leu88 (Figure 4(d)). S-HEPP has aromatic interactions with Phe91 and Phe84 and hydrophobic interactions with Leu88 (Figure S2B). S-Cl-HEPP establishes hydrophobic interaction with Val88, Phe91, and Leu88 (Figure S2C). S-F-HEPP binds by means of aromatic interactions with Phe84, Phe91, and Leu88 (Figure S2D).

Supplementary Table  2 lists the amino acids involved in the interaction of HEPP, F-HEPP, and Cl-HEPP in their absolute R and S configurations, as well as for phenytoin on the sodium channel Nav1.2.

## 4. Discussion

The design of new anticonvulsant compounds is a relevant topic in medicinal chemistry today. In the present study, we analyzed the effect of two derivatives of a compound previously reported as an anticonvulsant agent (HEPP). Whereas the mechanism of action of this lead compound is only partially understood, there is even less known about the mechanism of structurally related compounds.

As a validation of the current evaluations, the ED_50_ values of phenobarbital and sodium valproate (classically considered as reference drugs on these tests, Table 1) against seizures induced by MES and PTZ are within the 95% confidence limits reported in the literature [35–38]. The ED_50_ values obtained for these reference compounds as well as those found for HEPP, F-HEPP, and Cl-HEPP against the MES and PTZ seizure models also statistically correspond to the ED_50_ according to a verification test [19] that was applied.

As aforementioned, scant data exist about HEPP derivatives and especially in relation to the compounds tested in this study. Only limited anticonvulsant data were obtained from one seizure model. The International League Against Epilepsy (ILAE) recommends using at least the MES and PTZ seizure models for the initial selection of new anticonvulsant compounds [39]. In these seizure models, the ED_50_ values for the anticonvulsant activity of HEPP, F-HEPP, and Cl-HEPP showed that the addition of fluorine or chlorine atoms in the paraposition of the aromatic ring increases the anticonvulsant potency of the compounds compared to HEPP. In the verification test of Lorke's method modified by Garrido-Acosta et al. [19], a lower dose of F-HEPP or Cl-HEPP (versus HEPP) was required to protect 50% of the mice against seizures induced by MES or PTZ. Although the ED_50_ values were the same for F-HEPP and Cl-HEPP against seizures by PTZ (43.5 mg/kg), they were different for these two compounds against seizures induced by MES (87.1 mg/kg for F-HEPP and 62.0 mg/kg for Cl-HEPP). Hence, the effects resulting from the halogenation of HEPP demonstrate the importance of a slight modification of the lead compound by a substitution in the aromatic ring. Similarly, Mishra and Baker [40] found that a slight change in the lead compound 4-(1-hydroxy-2, 2, 2-trifluoroethyl)-phenyl with alkyl-bromide substitutions led to a difference in the anticonvulsant effects of the resulting molecules when tested in the seizure model of MES and PTZ.

Additionally, we performed a test of neurotoxicity for HEPP, F-HEPP, and Cl-HEPP with the rotarod test [20, 21]. Whereas twice the ED_50_ for HEPP (260 mg/kg) against MES was required to cause motor disparity, twice the ED_50_ for F-HEPP and Cl-HEPP against MES seizures did not induce this effect. Meza-Toledo et al. [12] reported a median toxic dose for HEPP of 214 mg/kg based on the rotarod test. The present results show that 260 mg/kg of HEPP reduced the endurance time on the rotarod by more than 50% (Figure 3). Overall, this evidence suggests that F-HEPP and Cl-HEPP are safer than HEPP.

The computational molecular docking studies provide data on the receptors involved in the anticonvulsant effects, allowing for the identification of possible patterns of ligand-receptor recognition for HEPP and its derivatives. This information should deepen our understanding of the mechanisms of action [41].

Thirteen amino acids in benzodiazepine binding site of GABA_A_ receptor have been previously reported (His101, Arg144, Gly157, Ala160, Thr162, Arg197, Gly200, Val202, Ser204, Ser205, Thr206, Tyr209, and Val2011). From this set of residues, the six amino acids thought to be of great importance for benzodiazepine recognition are His101, Arg144, Ser204, Ser205, Thr206, and Tyr209. Regarding flunitrazepam, Ser205 is considered a key residue [26, 42–44].

The present computational molecular docking studies show that HEPP, F-HEPP, and Cl-HEPP bind close to the benzodiazepine binding site, sharing interactions with 2 to 4 amino acids considered as being inside this site. However, these amino acids are not considered important for the efficacy of benzodiazepine (Figure 4, Supplementary Figure  1, and Supplementary Table  1). Thus, these results suggest that HEPP, F-HEPP, and Cl-HEPP may modulate the action of the GABA_A_ receptor by inducing conformational changes in the benzodiazepine binding site [28, 45] or by acting on another nearby site. This action could be complemented by an interaction of the phenyl alcohol of these amides on GABA_B_ [17]. Future studies should be carried out to confirm or discard the latter suggestion.

On the other hand, the protective effects of HEPP, F-HEPP, and Cl-HEPP on the MES seizure model suggest feasible interactions of these compounds mediated by the sodium channel [46, 47]. In this sense, HEPP, F-HEPP, and Cl-HEPP were studied on the sodium channel open pore [29] and the docking results were compared to the phenytoin interaction with this channel. The current docking studies reveal that phenytoin interacted with eight amino acids: Leu88, Asn84, Leu91, Ile87, Tyr91, Phe84, Ser83, and Val87. This result is in agreement with the findings of other works [41, 48]. Hence, the data obtained from docking and from the structural evaluation of this 3D channel model (not shown) validate the theoretical protocol herein employed.

Regarding the interactions of the tested compounds, we observed that 3 (of 6) amino acids of R-HEPP are shared with the predicted site for phenytoin. Of these, two are thought to be important for the binding site of phenytoin (Phe84E and Val87H). Interestingly, S-HEPP and the absolute configurations of F-HEPP and Cl-HEPP all occupy the same site of one derivate of phthalimide reported recently. This compound is more potent than phenytoin for blocking the sodium channel [41], which may be related to the higher potency of the tested compounds.

The experimental results of the MES seizure model and the binding energy in docking studies suggest that Cl-HEPP is more potent that F-HEPP and that both compounds have higher potency than HEPP. Thus, research pertaining to the phenyl alcohol of these amides suggests that they act as regulators on GABA_A_ receptors near to the benzodiazepine site. Furthermore, Cl-HEPP may act on the GABA_B_ receptor in other ways [14]. We suggest that these ligands may disrupt sodium channel conductance by reaching some residues that are key in the regulation of ion permeability.

## 5. Conclusions

By adding a fluorinated or chlorinated atom at the paraposition of the phenyl group of HEPP, the anticonvulsant potency was increased. The experimental potency in decreasing order was Cl-HEPP > F-HEPP > HEPP. Hence, the present results suggest a structure-activity relationship involving a halogen addition in the aromatic ring of the reference compound. These experimental results, supported by ligand-receptor docking studies, suggest that HEPP, F-HEPP, and Cl-HEPP act as regulators of GABA action and perhaps disrupt the sodium channel as well. Based on ILAE recommendations for the initial evaluation of anticonvulsant compounds, Cl-HEPP and F-HEPP are good candidates for preclinical studies. Moreover, these derivatives have an ED_50_ < 100 mg/kg and they did not show neurotoxic effects in the rotarod test at twice the ED_50_ against MES.

## Supplementary Material

Supplementary Material includes the docking results for (S)-configuration of tested ligands on GABAA receptor or the sodium channel Nav1.2, as well as the energy values and residues involved in the recognition for all tested ligands.

## Figures and Tables

**Table 1:** ED_50_ and confidence limits of HEPP and its fluorinated and chlorinated derivatives against MES and PTZ.

Treatments	MES mg/kg	C.L. 95%	PTZ mg/kg	C.L. 95%
HEPP	129.6	14% to 70%	66.4	14% to 70%
F-HEPP	87.1	14% to 70%	43.5	14% to 70%
Cl-HEPP	62.0	14% to 70%	43.5	22% to 78%
Sodium valproate	261.2	14% to 70%	159.7	22% to 78%
Phenobarbital	16.3	30% to 86%	12.7	14% to 70%

MES: maximal electroshock; PTZ: pentylenetetrazole; HEPP: 3-hydroxy-3-ethyl-3-phenylpropionamide; F-HEPP: 3-hydroxy, 3-ethyl, 3-(4′-fluorophenyl)propioanamide; Cl-HEPP: 3-hydroxy, 3-ethyl, 3-(4′-chlorophenyl)propionamide; C.L.: confidence limits of the expected fraction.
