# Supplementary material for: Anticonvulsant and Toxicological Evaluation of Parafluorinated/Chlorinated Derivatives of 3-Hydroxy-3-ethyl-3-phenylpropionamide
Source: Biomed Res Int. 2016 Feb 24;2016:3978010. doi: 10.1155/2016/3978010 (PMC4783531; doi:10.1155/2016/3978010)
Supplement: Supplementary file 1 — Supplementary Material includes the docking results for (S)-configuration of tested ligands on GABAA receptor or the sodium channel Nav1.2, as well as the energy values and residues involved in the recognition for all tested ligands. [file 3978010.f1.pdf]

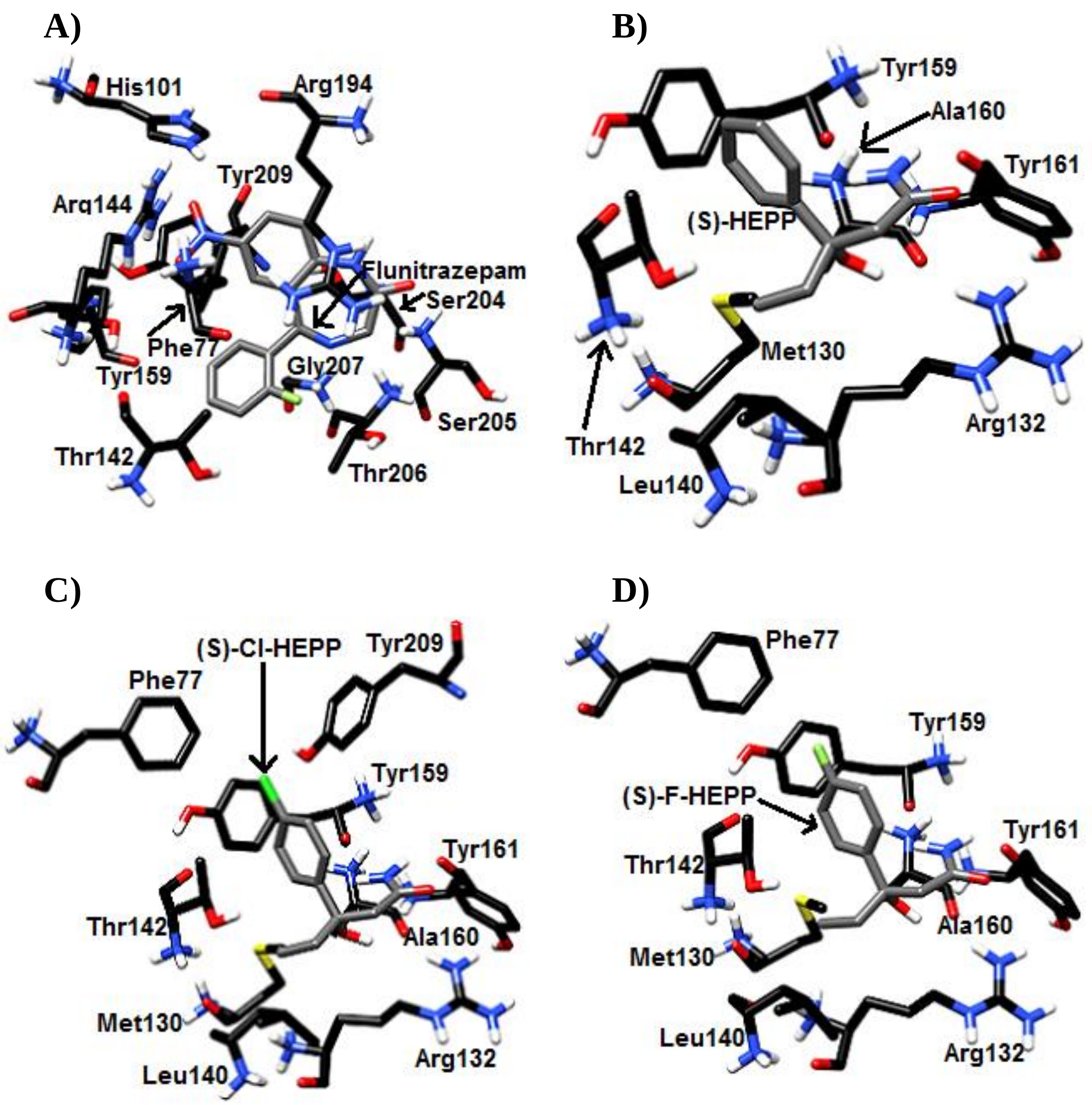

Supplementary Figure 1: Binding of flunitrazepam, (S)-HEPP, (S)-Cl-HEPP, and (S)-F-HEPP on the benzodiazepine binding site of $GABA_A$ receptor.

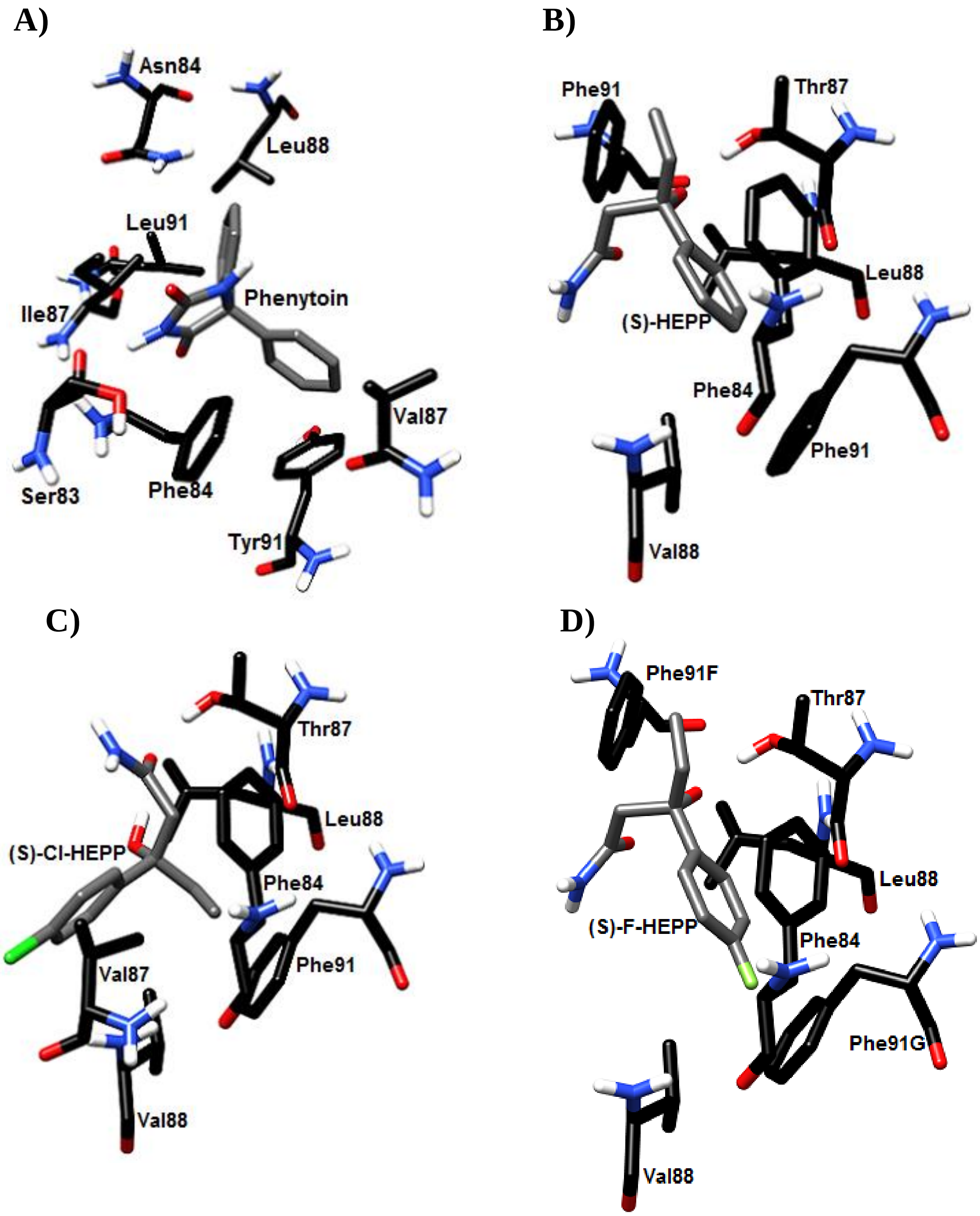

Supplementary Figure 2: Docking of phenytoin, HEPP and tested ligands on sodium channel Nav1.2.

Supplementary Table 1. Amino acids reached by the tested compounds to produce the interaction described in Figures 4 and Supplementary Fig. 1, and the binding free energies of HEPP, F-HEPP, and Cl-HEPP in docking studies on $GABA_A$ receptor.

Compound	Flunitrazepam	(R)-HEPP	(R)-Cl-HEPP	(R)-F-HEPP	(S)-HEPP	(S)-Cl-HEPP	(S)-F-HEPP
ΔG	-7.42	-5.28	-5.81	-5.3	-5.52	-6.08	-5.59
Amino acids of interaction	Phe77		Phe77	Phe77		Phe77	Phe77
	His101						
		Met130	Met130	Met130	Met130	Met130	Met130
		Arg132	Arg132	Arg132	Arg132	Arg132	Arg132
		Leu140	Leu140	Leu140	Leu140	Leu140	Leu140
	Thr142	Thr142	Thr142	Thr142	Thr142	Thr142	Thr142
	Arg144						
	Tyr159	Tyr159	Tyr159	Tyr159	Tyr159	Tyr159	Tyr159
		Ala160	Ala160	Ala160	Ala160	Ala160	Ala160
				Tyr161	Tyr161	Tyr161	Tyr161
	Arg194						
	Ser204						
	Ser205						
	Thr206						
	Gly207		Gly207				
	Tyr209		Tyr209			Tyr209	

(R)-HEPP: (R)-3-hydroxy, 3-ethyl, 3-phenylpropionamide,); (R)-Cl-HEPP: (3R)-3-hydroxy, 3-ethyl, 3-(4'-chlorophenyl) propionamide; (3R)-F-HEPP: (3R)-3-hydroxy, 3-ethyl, 3-(4'-fluorophenyl)propionamide; (S)-HEPP: (3S)-3-hydroxy, 3-ethyl, 3-phenylpropionamide; (S)-Cl-HEPP: (3S)-3-hydroxy, 3-ethyl, 3-(4'-chlorophenyl) propionamide; (3S)-F-HEPP: (3S)-3-hydroxy, 3-ethyl, 3-(4'-fluorophenyl)propionamide.

Supplementary Table 2: The amino acids reached by the tested compounds to produce the interaction described in Figures 5 and Supplementary Figure 2, and the binding free energies of HEPP, F-HEPP, and Cl-HEPP in docking studies on sodium channel Nav1.2.

Compound	Phenytoin	(R)-HEPP	(R)-Cl-HEPP	(R)-F-HEPP	(S)-HEPP	(S)-Cl-HEPP	(S)-F-HEPP
ΔG	-6.01	-4.47	-4.6	-4.37	-4.43	-4.47	-4.42
	Chain E	Chain E	Chain E	Chain E	Chain E	Chain E	Chain E
	Ser83*						
	Phe84*	Phe84					
	Ile87	Ile87					
		Asn88					
	Leu91	Leu91					
Amino acids of interaction	Chain F	Chain F	Chain F	Chain F	Chain F	Chain F	Chain F
	Asn84						
	Leu88*						
				Phe91	Phe91		Phe91
	Chain G	Chain G	Chain G	Chain G	Chain G	Chain G	Chain G
			Thr87	Thr87	Thr87	Thr87	Thr87
			Leu88	Leu88	Leu88	Leu88	Leu88
			Phe91	Phe91	Phe91	Phe91	Phe91
	Chain H	Chain H	Chain H	Chain H	Chain H	Chain H	Chain H
			Phe84	Phe84	Phe84	Phe84	Phe84
	Val87*	Val87	Val87			Val87	
		Val88	Val88	Val88	Val88	Val88	Val88
	Tyr91						

(R)-HEPP: (R)-3-hydroxy, 3-ethyl, 3-phenylpropionamide,); (R)-Cl-HEPP: (3R)-3-hydroxy, 3-ethyl, 3-(4'-chlorophenyl) propionamide; (3R)-F-HEPP: (3R)-3-hydroxy, 3-ethyl, 3-(4'-fluorophenyl)propionamide; (S)-HEPP: (3S)-3-hydroxy, 3-ethyl, 3-phenylpropionamide; (S)-Cl-HEPP: (3S)-3-hydroxy, 3-ethyl, 3-(4'-chlorophenyl) propionamide; (3S)-F-HEPP: (3S)-3-hydroxy, 3-ethyl, 3-(4'-fluorophenyl)propionamide.
*Amino acids relevant in the phenytoin binding [36].
